# Tuning the Phosphoryl Donor Specificity of Dihydroxyacetone Kinase from ATP to Inorganic Polyphosphate. An Insight from Computational Studies

**DOI:** 10.3390/ijms161126073

**Published:** 2015-11-24

**Authors:** Israel Sánchez-Moreno, Isabel Bordes, Raquel Castillo, José Javier Ruiz-Pernía, Vicent Moliner, Eduardo García-Junceda

**Affiliations:** 1 Departamento de Química Bioorgánica, Instituto de Química Orgánica General, CSIC. Juan de la Cierva 3, Madrid 28006, Spain; isra.sanchez@iqog.csic.es; 2 Departament de Química Física i Analítica, Universitat Jaume I. Castellón 12071, Spain; bordes@uji.es (I.B.); rcastill@uji.es (R.C.); pernia@uji.es (J.J.R.-P.)

**Keywords:** biocatalysis, computational chemistry, DHAP-dependent aldolases, dihydroxyacetone kinase, enzyme directed evolution, quantum mechanics/molecular mechanics

## Abstract

Dihydroxyacetone (DHA) kinase from *Citrobacter freundii* provides an easy entry for the preparation of DHA phosphate; a very important C3 building block in nature. To modify the phosphoryl donor specificity of this enzyme from ATP to inorganic polyphosphate (poly-P); a directed evolution program has been initiated. In the first cycle of evolution, the native enzyme was subjected to one round of error-prone PCR (EP-PCR) followed directly (without selection) by a round of DNA shuffling. Although the wild-type DHAK did not show activity with poly-P, after screening, sixteen mutant clones showed an activity with poly-phosphate as phosphoryl donor statistically significant. The most active mutant presented a single mutation (Glu526Lys) located in a flexible loop near of the active center. Interestingly, our theoretical studies, based on molecular dynamics simulations and hybrid Quantum Mechanics/Molecular Mechanics (QM/MM) optimizations, suggest that this mutation has an effect on the binding of the poly-P favoring a more adequate position in the active center for the reaction to take place.

## 1. Introduction

**Figure 1:**
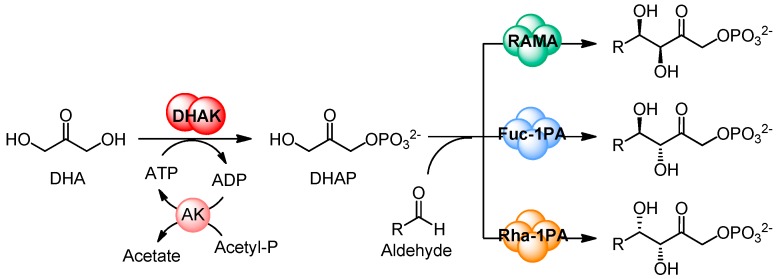
Multi-enzyme system for C–C bond formation catalyzed by DHAP-dependent aldolases, based in the *in situ* phosphorylation of DHA catalyzed by DHAK from *Citrobacter freundii* (*C. freundii*). DHA: Dihydroxyacetone; DHAK: dihydroxyacetone kinase; AK: acetate kinase; RAMA: rabbit muscle aldolase; DHAP: dihydroxyacetone phosphate.

Dihydroxyacetone (DHA) kinase from *Citrobacter freundii* (*C. freundii*) provides an easy entry for the preparation of DHA phosphate, a very important C_3_ building block in nature since it is used as phosphoryl donor in several enzyme-catalyzed aldol reactions [1]. Aldol addition reaction is one of the most useful tools that have the synthetic chemist for the construction of new C–C bonds [2,3,4]. Dihydroxyacetone phosphate (DHAP)-dependent aldolases are among the most important biocatalysts for C–C bond formation [5,6,7,8,9,10]. Their major synthetic advantage is that the stereochemistry of the two newly formed stereogenic centers is controlled by the enzymes and, moreover, the four DHAP-dependent aldolases are stereocomplementary; so, from two given substrates, it is possible to obtain the four diastereoisomers. However, it is also well known that their major drawback is their strict specificity for DHAP. Although several chemical and enzymatic routes of DHAP synthesis have been described in the literature, an efficient method of DHAP preparation is still essential [11]. In this sense, our research group has developed a straightforward multi-enzyme system for one-pot C–C bond formation catalyzed by DHAP-dependent aldolases, based in the use of the recombinant ATP-dependent DHA kinase from *C. freundii* CECT 4626, for *in situ* DHAP formation [12,13,14,15]. This enzyme has the highest described catalytic efficiency for DHA phosphorylation, about one order of magnitude higher than the rest of the enzymes that have been also used for the preparation of DHAP [16]. The multi-enzyme system was completed with the *in situ* regeneration of ATP catalyzed by acetate kinase (Figure 1). Thereafter, we have reported the engineering of a new bifunctional enzyme that displays both aldolase and kinase activities in the same protein [17,18].

**Figure 2:**
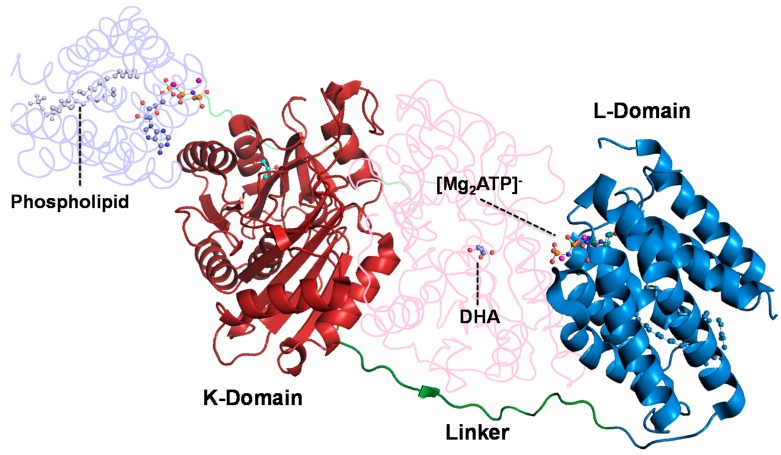
Crystallographic structure of DHAK from *C. freundii* strain DSM 30040 (pdb 1un9). In the N-terminal domain—K-domain—is where the DHA binding site is located. The ATP binding site is found in the C-terminal—l-domain.

Dihydroxyacetone kinase (DHAK) from *C. freundii* presents several features that make it a very interesting enzyme. It is a homodimer and each subunit is formed by two domains [19]. The K-domain is where the DHA binding site is located and in the l-domain is founded the ATP binding site (Figure 2).

In the dimer, the subunits are arranged in an anti-parallel way, therefore, the K-domain of one subunit is faced with the l-domain of the other subunit. The ATP binding domain is a barrel formed by eight amphipathic alpha-helix stabilized by a lipid. DHAK is the only kinase known to have an all-α nucleotide-binding domain representing a new fold group in the kinase classification scheme, as its fold is unlike any other kinase with known structure [20]. From the functional point of view, we have demonstrated that this enzyme presents a promiscuous activity [21]. Besides the phosphorylation of DHA, DHAK is able to catalyze, in the same active center, the cyclization of flavin adenine dinucleotide (FAD) to cyclic flavin mononucleotide (FMN). This catalytic promiscuity is modulated by the divalent cation that forms the complex with the phosphorylated substrate. The manganese concentration acts as a switch to turn on or off the kinase (natural) or the cyclase (promiscuous) activity.

One disadvantage of the multi-enzyme system described above is the need for ATP regeneration. To eliminate the ATP regeneration system, it is necessary to found a phosphoryl donor cheaper than ATP and, more importantly, that the final product does not inhibited the kinase. A good candidate it is the inorganic polyphosphate (poly-P). Inorganic polyphosphate (poly-P) is a linear polymer of up to hundreds of orthophosphate (P_i_) residues linked by high-energy phosphoanhydride bonds [22,23,24,25,26]. A large amount of poly-P is routinely produced as sodium hexametaphosphate (about 13 to 18 phosphate residues) for food additives and other industrial uses, making it a phosphoryl donor inexpensive compared to ATP, acetyl-P and phosphoenolpyruvate (PEP). In addition, poly-P usually does not promote inhibition on phosphotransferases [27].

Nowadays, Computational Chemistry has emerged as a powerful tool for the analysis of reaction mechanisms in complex environments such as enzyme-catalyzed processes and the information obtained from these studies provides clues to guide the development of new and more efficient biological catalysts [28,29,30,31]. Therefore, a thorough computational based characterization of active mutants, by comparison with the wild-type enzyme, could provide information about the evolution of the substrate-protein interactions and, consequently, on the origin of the measurable activities. The results can also be used to deduce which residues of the active site are responsible for the preferential stabilization of the complex and thus, using an active mutant as starting point, to design a new catalyst capable of enhancing the rate constant of the chemical step. Herein, we describe the results obtained after a round of error-prone PCR (EP-PCR) followed (without previous selection) by a round of DNA shuffling. Theoretical calculations, based on molecular dynamics (MD) simulations and hybrid Quantum Mechanics/Molecular mechanics (QM/MM) optimizations, have been performed to study the effects of mutations on the most active mutant (Glu526Lys). A proposal of the origin of the activity will be then based on the simulations.

## 2. Results and Discussion

### 2.1. Substrate Specificity of the Wild-Type DHAK

Previously, to begin our program of Directed Evolution, we analyzed the substrate specificity of the wild-type DHAK towards the phosphoryl donor. Kinase activity of the enzyme was assayed with the five natural nucleoside triphosphates, ATP, GTP, CTP, TTP and UTP; inorganic triphosphate; and poly-P. Only ATP was substrate of the enzyme, displaying a catalytic efficiency (*k*_cat_/*K*_M_) of 6.9 × 10^4^ s^−1^·M^−1^ (*K*_M_ 3.5 × 10^−4^ ± 0.01 mM and *k*_cat_ 24.02 ± 0.37 s^−1^). None of the other phosphoryl donors assayed showed activity with the wild-type DHAK, at least in the limit of detection of our assay.

### 2.2. Generation of a DHAK Mutant Library

**Figure 3:**
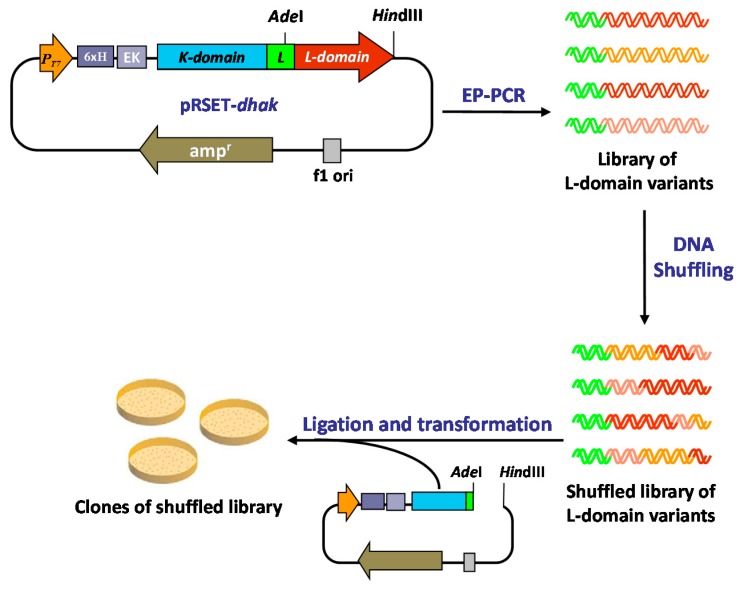
Directed evolution approach applied to modify the phosphoryl donor specificity of the DHAK from *C. freundii*.

Given the particular structure of the DHAK (two domains connected by an 18 amino acids linker; Figure 2), we envisioned a “cassette” strategy in which it is possible to take off only the ATP-binding domain and carry out on it the evolution process, keeping the DHA-binding domain unmodified. To accomplish this strategy, we took advantage of the presence in the linker region of a unique restriction site for the endonuclease *Ade*I that allow to cut-off from the pRSET-*dhak* plasmid, the l-domain where the ATP binding site is located (Figure 3).

To introduce genetic diversity into the sequence of the l-domain, we performed a round of EP-PCR followed directly—without previous selection—by a round of DNA shuffling. The 620 pb *domL* fragment was amplified under standard EP-PCR conditions [32,33,34], optimized to introduce 2–3 mutations per kilobase. The mutation rate (2.9 bases change/Kb) was verified by DNA sequencing of six randomly picked colonies of the l-domain library. The analysis of these sequence showed that DNA polymerase used in the PCR reaction (*Taq* pol) is more likely to mutate AT (67%) than GC (33%). These results were in agreement with the mutational tendency previously described in the literature [35]. The library of l-domain variants obtained in the EP-PCR step was carefully digested with DNase I, and fragments in the range of 50–100 bp were selected. The purified fragments were reassembled in a PCR reaction without primers. The number of reactions cycles (60) and the amount of DNA used as template (20% *v*/*v*, see Experimental Section) were optimized to obtain PCR products with an average size closed to full-length of the l-domain sequence (see Experimental Section).

Finally, the purified full-length l-domain sequences were amplified using standard PCR with specific primers and shuffled library of ATP-binding domains was re-introduced in the pRSET-*dhak* plasmid, restoring the whole *dhak* gene.

### 2.3. Screening of the DHAK Mutant Library

**Figure 4:**
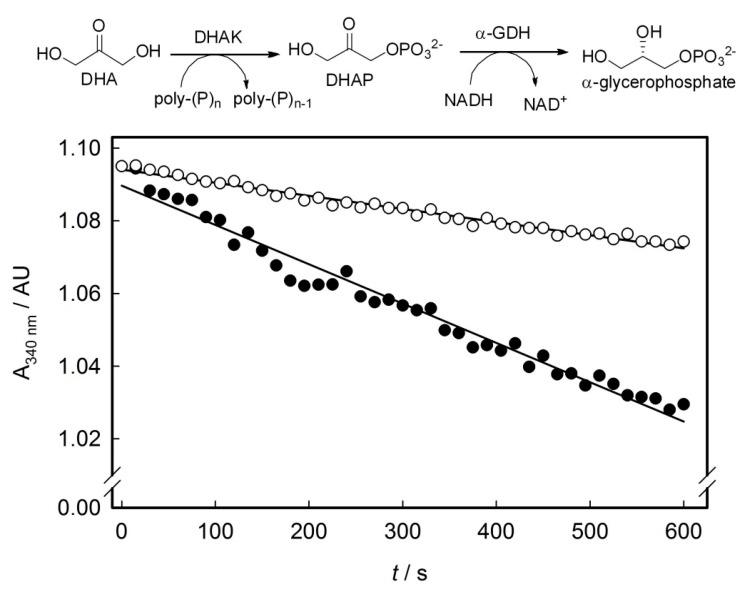
Enzymatic assay used to evaluate the poly-P-dependent kinase activity in the DHAK mutant library and time-dependent decrease of NADH absorbance at 340 nm corresponding to the mutant 3E4. (○) Unspecific NADH oxidation measured in absence of DHA and (●) slope obtained in presence of DHA. For details, see Section 3.6.

The mutant library was screened for their ability to phosphorylate DHA using poly-P as phosphoryl donor. Activity was measured by a coupled enzymatic assay, in which DHAP formed during the reaction in presence of poly-P was reduced to α-glycerophosphate with concomitant oxidation of NADH to NAD^+^. Assay samples were prepared on 96 well plates and time-dependent decrease of NADH absorbance at 340 nm was spectrophotometrically monitored. In the primary screening, the activity was assayed in cell free extracts (CFE) from each clone of the shuffled library. Unspecific NADH oxidation was measured in reaction mixtures lacking DHA and subtracted to the slope (activity) obtained in presence of DHA (Figure 4). Thus, the resulting slope should be proportional to the kinase activity using poly-P as phosphoryl donor substrate. A control population integrated by clones expressing the wild-type DHAK was analyzed following the same screening process. Both the mutant (*M*) and the control (*C*) populations followed a normal distribution according to the Kolmogorov–Smirnov test for normality [36]. The standard normal distribution of poly-P dependent activity in population *M* with respect to population *C* was obtained calculating the standard activity *Z* for each of the individuals of *M* according Equation (1).

(1) $$Z=\frac{\left(x_{M}-\mu_{M}\right)}{\sigma_{c}}$$

**Figure 5:**
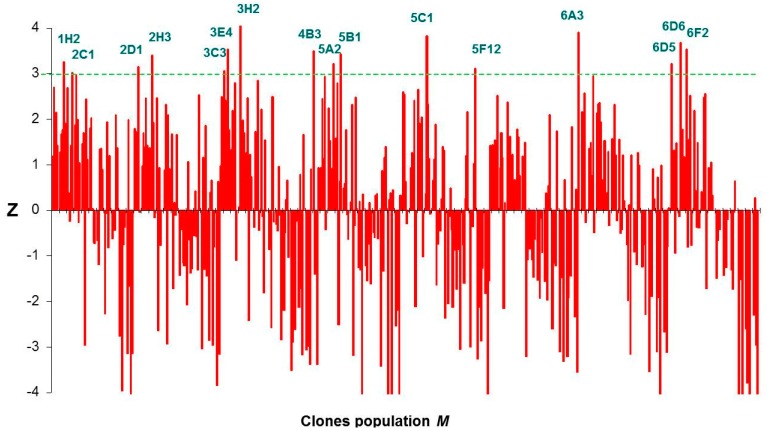
Graphic representation of the *Z*-scores of DHAK mutant population *M*.

The statistical parameter *Z* tells us how much and in what direction—above or below—is away from the mean of the mutant population (*μ_M_*) each individual value of slope (*x_M_*) in the population *M* with respect to the standard deviation (*σ_C_*) of the slope values in the control population *C*. Thus, clones with a value of *Z* ≥ 3, had a probability ≥98% that the slope measured was greater than the mean of the slope values in the control population. After carrying out the statistical analysis, we found 16 clones with *Z* ≥ 3 (Figure 5). Although the statistical analyses employed returned an extremely high number of positives, we decided to express and purified the 16 positive mutants identified. The expression of the putative DHAK mutants was carried out as described in the Experimental Section and analyzed by SDS-PAGE. In all cases, a band of about 63 kDa corresponding to DHAK weight could be detected in the CFE, indicating that the mutant proteins were expressed soluble. Activities of the 16 DHAK mutants were determined after purification by affinity chromatography (IMAC). In seven of the mutants identified as positive in the statistical analyses (2D1, 3C3, 3H2, 5C1, 6A3, 6D5 and 6D6), it was impossible to detect any kinase activity using poly-P as phosphoryl donor. Another group of mutants (2C1, 2H3, 4B3, 5A2, 5B1 and 6F2) was barely in the limit of detection of the employed assay. Finally, three mutants—1H2, 3E4 and 5F12—were clearly positive with polyphosphate-dependent kinase activities in the order of 10 mU per mg of protein (see Table S1). The mutant 1H2 showed the highest activity with poly-P (13.4 mU per mg of protein). This mutant presented a specific activity with ATP of 9.2 U per mg of protein, similar to the specific activity showed by the wild-type DHAK (10.7 U per mg of protein) and three orders of magnitude higher than the activity showed with poly-P.

### 2.4. Computational Studies of the Wild-Type and the Mutant 1H2

According to the experimental results, the computational studies were focused on the wild-type and the most active mutant: the 1H2 that presents only the mutation Glu526Lys, located on a flexible loop of the l-domain, in the surrounding of the ATP-binding site. This substitution could suggest a stronger electrostatic interaction between the positively charged lysine and the negatively charged poly-P. Nevertheless, our MD simulations suggest that mutation does not have any direct effect on the distance between the poly-P and the amino acid on position 526.

**Figure 6:**
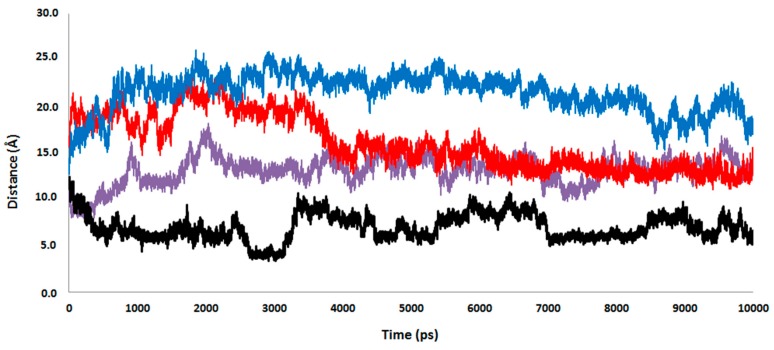
Time evolution of the distances between the closest atom of poly-P and the magnesium atoms in the wild-type (purple line), the magnesium atoms in the 1H2 mutant (black line), the residue 526 in the wild-type (red line), and the residue 526 in the 1H2 mutant (blue line). Results obtained from the 10 ns MD simulation.

**Figure 7:**
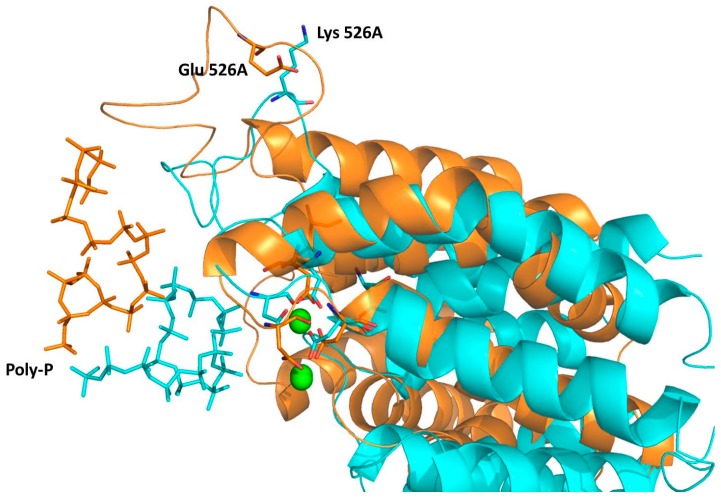
Overlapping of representative snapshots of the average structures in the last 500 ps of the MD simulations obtained with the wild type (gold) and 1H2 mutant (blue) complexed with the poly-P. Magnesium cations are represented as green balls. Poly-P docked in the l-domain of the 1H2 mutant (blue) and wild-type (gold) DHAK are shown as sticks. The Lys that substitutes to the Glu in position 526 in the mutant, and aspartate residues that interact with ATP in the wild-type DHAK, are also shown as sticks.

As observed in Figure 6, this distance is even shorter in the wild type than in the 1H2 (see red and blue lines, respectively). Interestingly, replacing the Glu residue on position 526 with a Lys provokes, instead, a significant approximation of the poly-P to the active site of the protein. Thus, despite both wild-type and 1H2 MD simulations starting from the same geometry, a significantly shorter distance between the closest phosphate group of poly-P and any of the Mg cations is reached after 500 ps in the 1H2 than in the wild-type. Thus, it seems that poly-P is better accommodated into the 1H2 mutant active site than in the wild type, in agreement with an experimentally observed higher catalytic activity. This effect can be shown on Figure 7, where an overlapping of both systems after the MD simulations, centered in the Mg cations, is presented. Apparently an indirect effect is produced under mutation of the protein since, despite a movement of the loop containing residue 526, no approximation is observed between the negatively charged poly-P and the new positively charged Lys 526. In fact, poly-P appears to be closer to the residue 526 in the wild-type than in the mutant, as indicated in Figure 6. This is probably due to a bending of the poly-P that favors the approximation to the active site while the new cation preferentially interacts with the solvent.

**Figure 8:**
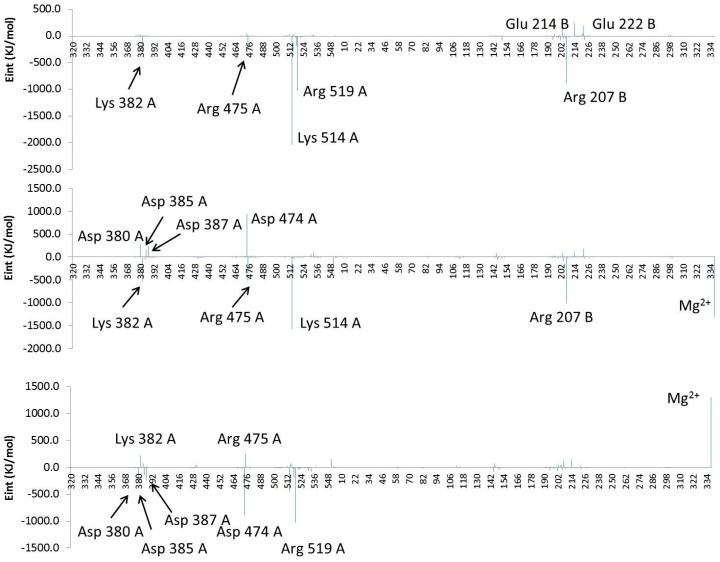
Contribution of individual amino acids to (poly-P)-protein interaction energy (Eint, in kJ·mol^−1^) for the wild type (**top panel**), 1H2 mutant (**middle panel**) and differences between both systems (**bottom panel**). Residues are ordered according to the sequence, and the Mg^2+^ ions are added at the end***.*** Results derived from the QM–MM interaction term of the QM/MM optimizations.

In order to confirm and quantify the interaction energy established between the poly-P and both proteins, 10 structures were selected from the MD trajectories to perform QM/MM optimizations. Keeping in mind that no covalent bond is established between the poly-P and the protein, the QM-MM interaction energy term can be directly related with the interaction between protein and poly-P. The average value of QM–MM interaction energy term of the 10 selected structures of the wild-type and mutant were −5100 ± 1100 and −7900 ± 1600 kJ·mol^−1^, respectively. According to these results, and although an expectedly large dispersion is obtained in potential energy values, the mutation has a clear effect in increasing the binding energy. The origin of the enzyme-(poly-P) interaction can be analyzed by decomposing the total interaction energy in a sum of contributions per residues. The averaged interaction energies, electrostatic plus van der Waals, of individual residues and the two magnesium cations of both systems with the poly-P is depicted in Figure 8. Bottom panel of Figure 8 shows the difference between the values obtained in the wild-type and the 1H2 mutant. Then, residues presenting positive values on this figure mean that they interact better with the mutant than with the wild-type. As observed, there are some residues, such as Lys382A, Arg475A, Lys514A and Arg207B, which present favorable interactions on both systems. On the contrary, Arg519A is stabilizing the complex only in the wild-type while the interaction of the poly-P with Mg^2+^ cations is enhanced after mutant. A graphical comparison of both systems, shown in bottom panel of Figure 8, suggests that the increase in the activity if 1H2 can be due to an enhancement of the interactions with Lys382, Arg475 and, in a dramatic way, with the magnesium cations.

On the other hand, non-favorable interactions with Asp380A, Asp385A, Asp387A and Asp474A, and the loss of the originally favorable interaction observed with Arg519A in the wild-type, are observed after mutation. It is important to point out that approximation of the negatively charged poly-P to the active site is ineludible associated with a repulsive interaction with the aspartate residues (especially those anchoring the Mg^2+^ cations and, in particular Asp380A, Asp385A and Asp387A).

**Figure 9:**
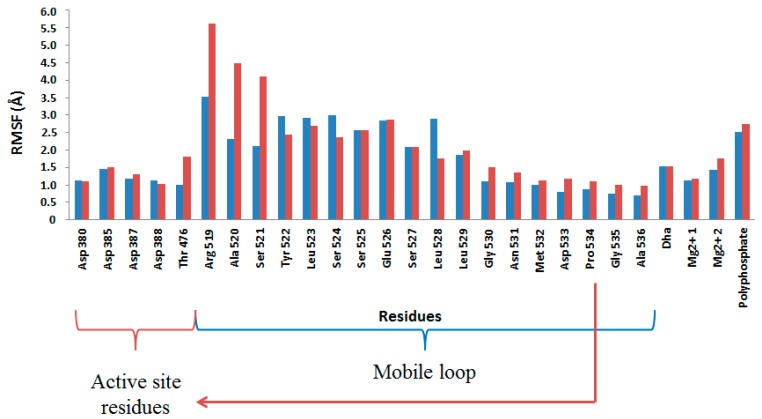
Root Mean Square Fluctuation (RMSF, in Å) of key residues computed from the last 5 ns of the MD simulation on the wild-type (blue bars) and 1H2 mutant (red bars).

Finally, analysis of the Root Mean Square Fluctuation (RMSF) of the key residues computed from the last 5 ns of the MD simulation on the wild-type and 1H2 mutant show that the later present a greater mobility, especially on the flexible loop, what could be related with the better accommodation of the poly-P. In particular, Arg519 mobility, together with Ala520 and Ser521, significantly increases after mutation of residue 526 (Figure 9). Therefore, further efforts to improve the phosphoryl donor specificity of DHAK from ATP to an inorganic poly-P should be focused in residues around Arg519 in the protein sequence. Finally, it is important to point out that these conclusions derived from the interactions that are important for poly-P fixation should be further tested by studying the next step of the catalytic process; the chemical reaction.

## 3. Experimental Section

### 3.1. Materials and General Procedures

UV/Visible spectra were recorded on a Spectra Max Plus 384 spectrophotometer at 25 °C. SDS-PAGE was performed in a Mini-Protean 3 Cell Electrophoresis Unit (BioRad, Hercules, CA, USA) using 10% and 5% acrylamide in the separating and stacking gels, respectively. Gels were stained with Coomassie brilliant blue R-250 (Applichem GmBH, Darmstadt, Germany). Electrophoreses were always run under reducing conditions, in the presence of 5% β-mercaptoethanol. Protein and DNA gels were quantified by densitometry using GeneGenius Gel Documentation and Analysis System (Syngene, Cambridge, UK) equipped with the analysis software GeneSnap and GeneTools (Syngene, Cambridge, UK). α-GDH and TIM were purchased from Sigma-Aldrich (St. Louis, MO, USA). Restriction enzymes, *Taq* polymerase and T4-DNA ligase were purchased from MBI Fermentas AB (Vilnius, Lithuania). *Citrobacter freundii* CECT 4626 was provided from the Spanish Type Culture Collection (Valencia, Spain). *E. coli* BL21 (DE3) competent cells were purchased from Stratagene Co. (San Diego, CA, USA). PCR primers were purchased from Isogen Life science (Utrecht, The Netherlands) and the pRSET-A expression vector was purchased from Invitrogen Co. (Carlsbad, CA, USA). IPTG was purchased from Applichem GmBH (Darmstadt, Germany). Plasmids and PCR purification kits were from Promega (Madison, WI, USA) and DNA purification kit from agarose gels was from Eppendorf (Hamburg, Germany). Nickel-iminodiacetic acid (Ni^2+^-IDA) agarose was supplied by Agarose Bead Technologies (Madrid, Spain). All other chemicals were purchased from commercial sources as reagent grade.

### 3.2. Error-Prone PCR Generated Library

The *dhak* gene encoding DHAK was cloned in the pRSET expression vector as previously described [21]. EP-PCR was performed on the 620 pb *dhak* fragment encoding the l-domain of DHAK using the following specific primers: leftward primer, 5′-GCGTAGCGCACGCGT-3′ (*Ade*I site underlined) and rightward primer, 5′-TTCTAAAGCTTTTAGCCCAGCTCACT-3′ (*Hind*III site underlined). The reaction mixture was prepared in a final volume of 100 μL containing 1 μM of each primer, dNTPs (0.2 mM each), 3.0 mM MgCl_2_, 50 mM KCl, 0.2 mM MnCl_2_, 1% DMSO, and 0.5 U *Taq* polymerase. EP-PCR was carried out using the following program: 94 °C for 2 min (1 cycle); 94 °C for 1.0 min, 55 °C for 1.0 min, 72 °C for 42 s (40 cycles); and 72 °C for 10 min (1 cycle). To verify the obtained mutation rate, a part of the l-domain variants library of was cloned in the plasmid pGEM^®^-T Easy and transformed in *E. coli* DH5α cells.

### 3.3. In Vitro Recombination by DNA Shuffling

DNA shuffling was done according to the method of Stemmer [37,38]. The pool of l-domain variants obtained by EP-PCR was digested during 5 min at RT with 0.14 U of DNase I in Tris-HCl buffer (50 mM; pH 7.4) containing MgCl_2_ 1 mM. DNA fragments in the range of 50–100 bp were purified from the agarose gel. After that, 20% *v*/*v* of the purified DNA fragments were recombined in a PCR reaction without primers containing dNTPs (0.4 mM each), MgCl_2_ (3.0 mM) and *Taq* polymerase (0.5 U). The thermal cycling program was: 94 °C for 2 min (1 cycle); 94 °C for 1.0 min, 55 °C for 1.0 min, 72 °C for 42 s (60 cycles); and 72 °C for 10 min (1 cycle). Finally, after 1:100 dilution of this primerless PCR product into a PCR mixture containing the specific primers described above, a single product of 620 bp corresponding to the length of the l-domain was amplified using 40 additional cycles. The resultant shuffled library of l-domain variants was ligated into the plasmid pRSET-*dhak*, previously digested with the endonucleases *Ade*I and *Hind*II. The digested plasmid contained the gene sequence encoding K-domain of the protein, which was necessary for restore the complete size of *dhak* gene in conjunction with l-domains variants. The resultant plasmids were transformed in *E. coli* BL21 (DE3) yielding over 1000 clones in a first generation.

### 3.4. Screening of DHAK Mutant Library

Masterplates were replicated twice into 96-well plates containing LB medium (150 μL) with ampicillin (2.5 μg/mL) and incubated at 37 °C with stirring at 200 rpm. The plates were sealed to prevent evaporation and after 90 min, protein expression was induced by addition of 0.5 mM of isopropyl-β-d-thiogalactopyranoside (IPTG). Plates were incubated in the same conditions for 14 h. Cells were harvested by centrifugation (5 min at 3000 rpm). Each cell pellet was dispersed in Tris-HCl buffer (50 mM; pH 8.0) and 10 μL of a solution containing EDTA (50 mM; pH 8.2) and lysozyme (0.35 mg/mL) were added. The plates were gently stirred at RT over 1 h and after they were kept at 4 °C overnight. Plates were centrifuged at 3000 rpm for 5 min and the cell free extracts (CFE) were transferred to new 96-well plates to carry out the activity assay. Screening was based on DHAK activity assay previously described [21] but using poly-P as phosphoryl donor instead of ATP. In this assay, DHAP formed by phosphorylation of DHA with poly-P is reduced to α-glycerophosphate with concomitant oxidation of NADH to NAD^+^ and time-dependent decrease of absorbance at 340 nm due to NADH oxidation is spectrophotometricaly monitored. To carry out the assay, to each well containing 15 μL of CFE, was added 0.3 mL of reaction mixture containing Tris-HCl (40 mM; pH 8.0), DHA (2.5 μM), NADH (0.2 μM), poly-P (150 μM), MgSO_4_ (2.5 μM) and αGDH (2.6 U).

### 3.5. Expression and Purification of Selected Mutants

**Figure 10:**
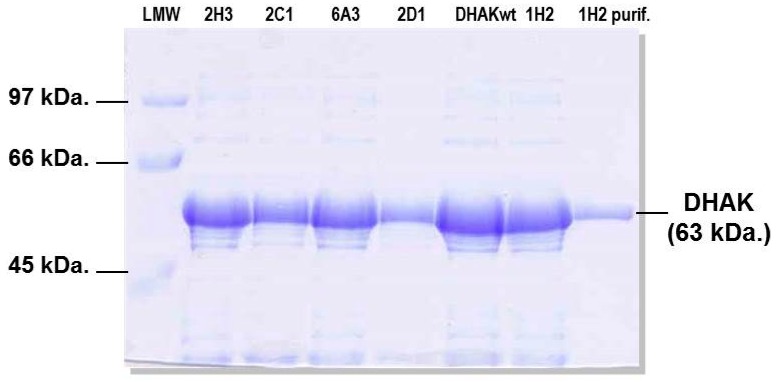
SDS-PAGE analysis of the expression of five mutants and the wild-type DHAK (DHAKwt). Left lane shows mutant 1H2 after purification.

Expression of wild-type DHAK and the 16 positive mutants was performed as previously described [21]. Expression was induced by IPTG addition (1 mM) when the culture reached an O.D_600nm_ of 0.5–0.6. After induction the culture temperature was dropped to 30 °C. The culture was maintained O/N and then centrifuged at 10,000× *g* during 10 min at 4 °C. The resulting pellet was treated with lysozyme and DNase for protein extraction. Recombinant proteins containing an N-terminal 6xHis tag was purified in a Ni^+2^-IDA-agarose column pre-equilibrated with sodium phosphate buffer (20 mM; pH 7.5). Protein elution was achieved with the same buffer containing imidazole 0.25 M. Mutant 1H2 was further purified by size-exclusion chromatography on a HiLoad 26/60 Superdex 75 PG column controlled using the AKTA-FPLC system (GE Healthcare Life Science, Little Chalfont, UK). Purification was carried out in 50 mM phosphate buffer pH 7.2 containing NaCl (0.15 M) at a constant flow rate of 1.0 mL/min (Figure 10).

### 3.6. Enzyme Activity Assays of Purified Mutants and wtDHAK

Phosphorylation of DHA was measured spectrophotometrically in a coupled enzymatic assay based in the reduction of DHAP to α-glycerophosphate, catalyzed by αGDH with concomitant oxidation of NADH to NAD+. The assays were run at 25 or 37 °C following the decrease of absorbance at 340 nm ($\varepsilon_{NADH}^{340}$ = 6220 cm^−1^·M^−1^) for 10 min in reaction mixtures of 1 mL containing Tris–HCl (40 mM, pH 8.0), DHA (2.5 μmol), NADH (0.2 μmol), ATP (5.0 μmol), MgSO_4_ (5.0 μmol), αGDH (1.26 U), TIM (12.6 U) and DHAK. When different phosphoryl donors were assayed, ATP was substituted by GTP, CTP, TTP, UTP, inorganic triphosphate or poly-P. Control activity assays were carried out with every mutant enzyme where the phosphoryl donor or DHA were not initially included. Also, blanks without enzyme were performed to be sure that no activity is observed when the enzyme is absent. After addition of the corresponding substrate, differences in the slopes were measured (Figure S1). One unit of kinase activity was defined as the amount that produces 1 μmol of DHAP per min under the above conditions.

### 3.7. Computational Model and Methods

The first step of our protocol was setting up the system for performing the MD simulations. The initial coordinates of the protein and the phospholipid were taken from the X-ray structure of the apo form of DHAK from *C. freundii* strain DSM 30040 (pdb 1UN8) [19]. Some missing residues located on the flexible loop of the l-domain were manually added within the help of Molden program [39]. In particular, and since the PDB file contained the full sequence of the protein, the missed residues were introduced in Molden together the 15 upstream and downstream residues in order to obtain the most favorable conformation from the missing loop. Anyway, as explained below, MD simulations will be run to explore different conformation of this flexible loop. The coordinates of DHA and magnesium cations were taken from the PDB file 1UN9 that corresponds to the Dha/ANP form [19]. Later, since the standard pKa values of ionizable groups can be shifted by local protein environments, an accurate assignment of the protonation states of all these residues at pH = 7 was carried out. Recalculation of the pKa values of the titratable amino acids has been done using the empirical PropKa program of Jensen *et al.* [40,41]. According to these results, all residues were found at their standard protonation state in aqueous solution, except His404 that was double protonated. Then, a 16 monomers poly-P was docked into the active site of the protein by placing a phosphate group in a position equivalent to the one the ATP would present for the chemical reaction to take place. Afterwards, in order to avoid overlapping between the poly-P and the protein, the former was displaced following a perpendicular vector. Then the mutated enzyme was generated by replacing Glu526 by a Lys. The 30 and 28 counter ions (Na^+^) were placed into optimal electrostatic positions around the wild type protein and mutant, respectively, in order to obtain electro neutrality. The systems were placed in a box of pre-equilibrated waters (100 Å × 80 Å × 80 Å), using the center of mass of the poly-P as the geometrical center. Any water with an oxygen atom lying in a radius of 2.8 Å from a heavy atom of the protein or counter ions was deleted. Afterwards, series of optimization algorithms (steepest descent conjugated gradient and L-BFGS-B) were applied. The two systems were equilibrated by means of 100 ps of Langevin–Verlet MD (NVT) [42] at temperature of 300 K using the fDYNAMO library [43]. Finally, 10 ns of MD simulations were performed within the NAMD parallel molecular dynamics code [44]. During the MD simulations, the atoms of the protein and poly-P were described using the OPLS-AA [45,46] force field while TIP3P force field [47] was used for the water molecules. Cutoffs for the nonbonding interactions were applied using a switching function, within a radius range from 14.0 to 16.0 Å, employing in all the simulations periodic boundary conditions. The analysis of the total energy and the RMSD of the wild-type and the mutant shows that both systems can be considered as equilibrated after the MD simulations (see Figures S2 and S3). Moreover, the DHA appears to be well fitted in the active site, with a pattern of interactions with the residues of the active site that was already observed in the initial X-ray diffraction structure (see Figure S4).

After the MD simulations, ten different structures from the last 5 ns of the two MD trajectories were selected to perform full hybrid QM/MM optimizations with fDYNAMO library. Treating the poly-P quantum mechanically and the protein molecular mechanically has the advantage of the inclusion of quantum effects such as poly-P polarization upon binding. Moreover, since no covalent bonds are established between the poly-P and the protein, interaction energy (that can be decomposed by residue) fits with the QM–MM interaction energy term of the full Hamiltonian and, consequently, the reported interaction energies include electrostatic and van der Waals terms computed quantum mechanically. Moreover, as the largest part of the system is described classically, enough sampling can be obtained at reasonable computational cost. All in all, a deeper understanding of the binding process can be obtained from more reliable results. In these calculations, the poly-P was treated by the semi-empirical AM1 [48] Hamiltonian, while the protein and water molecules were treated by OPLS-AA and TIP3P force fields, respectively.

## 4. Conclusions

Using an approach of Enzyme Directed Evolution, we have been able to generate a new activity on the DHAK from *C. freundii*. After a round of error-prone PCR followed by a round of artificial recombination by DNA shuffling, it was possible to detect a mutant showing kinase activity using poly-P as phosphate group donor in the order of tens of mU. This mutant, termed 1H2, presents only one mutation consisting of the change of Glu526, located in an unstructured loop in the surrounding of the ATP binding site, to a Lys. Computational studies allow concluding that this mutation does not increase the interaction of the new positively charged amino acid with the negatively charged poly-P. Instead, an indirect effect is generated that promotes a displacement of the loop that pushes the l-domain α -helix allowing the poly-P coming closer to the ATP binding site and then favoring the reaction to take place.

## Supplementary Materials

Supplementary materials can be found at http://www.mdpi.com/1422-0067/16/11/26073/s1.
