# Supplementary material for: Tuning the Phosphoryl Donor Specificity of Dihydroxyacetone Kinase from ATP to Inorganic Polyphosphate. An Insight from Computational Studies
Source: Int J Mol Sci. 2015 Nov 24;16(11):27835–49. doi: 10.3390/ijms161126073 (PMC4661931; doi:10.3390/ijms161126073)
Supplement: Supplementary file 1 [file ijms-16-26073-s001.pdf]

Supplementary Materials: Tuning the Phosphoryl Donor Specificity of Dihydroxyacetone Kinase from ATP to Inorganic Polyphosphate. An Insight from Computational Studies

Israel Sánchez-Moreno, Isabel Bordes, Rocío Martín-Hoyos, Raquel Castillo, José Javier Ruiz-Pernia, Vicent Moliner and Eduardo García-Junceda

Table S1. Polyphosphate-dependent kinase activities of the wild-type and mutant enzymes [a].

**Enzyme**	**wtDHAK**	**1H2**	**2C1**	**2D1**	**2H3**	**3C3**	**3E4**	**3H2**	**4B3**
Specific activity (mU·mg^{-1})	n.d [b]	13.4	2.6	n.d	1.9	n.d	7.7	n.d	0.1
**Enzyme**	**-**	**5A2**	**5B1**	**5C1**	**5F12**	**6A3**	**6D5**	**6D6**	**6F2**
Specific activity (mU·mg^{-1})	-	3.0	2.7	n.d	10.5	n.d	n.d	n.d	0.4

[a] Nomenclature of mutant enzymes is arbitrary and corresponds to the coordinates in the micro-plate; [b] n.d = not detected.

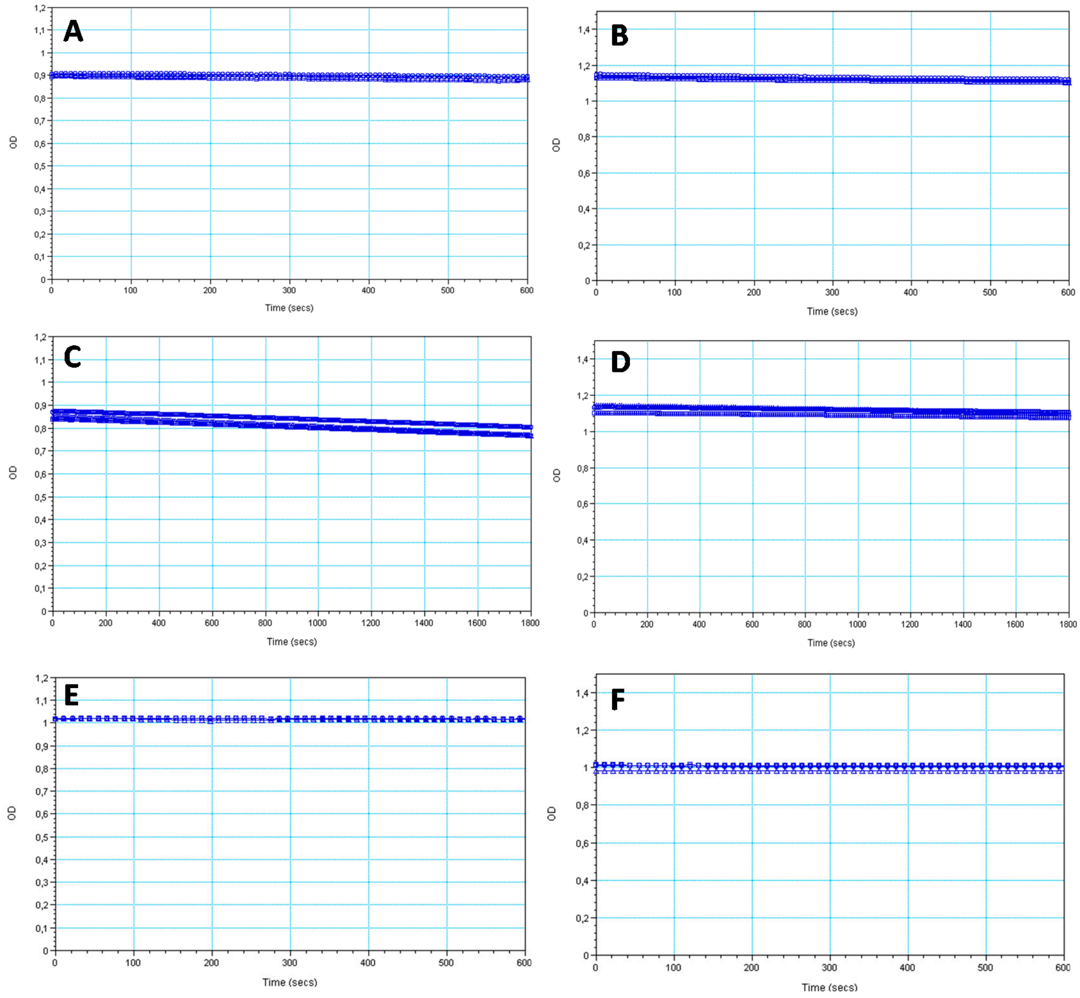

Figure S1. Absorbance at 340 nm was recorded along the time in triplicate reactions containing poly-P and mutant 1H2 (**A**); The same assay was performed with wild type DHAK enzyme (**C**); When DHA was added into the pre-incubated reactions, significant slope differences were observed in 1H2 mutant reactions (**B**) but no in DHAK wt ones (**D**); In order to exclude any spontaneous chemical phosphorylation, control reactions were incubated in absence of the corresponding enzyme: poly-P/DHA mixture without 1H2 mutant (**E**);and ATP/DHA mixture without DHAK wt (**F**).

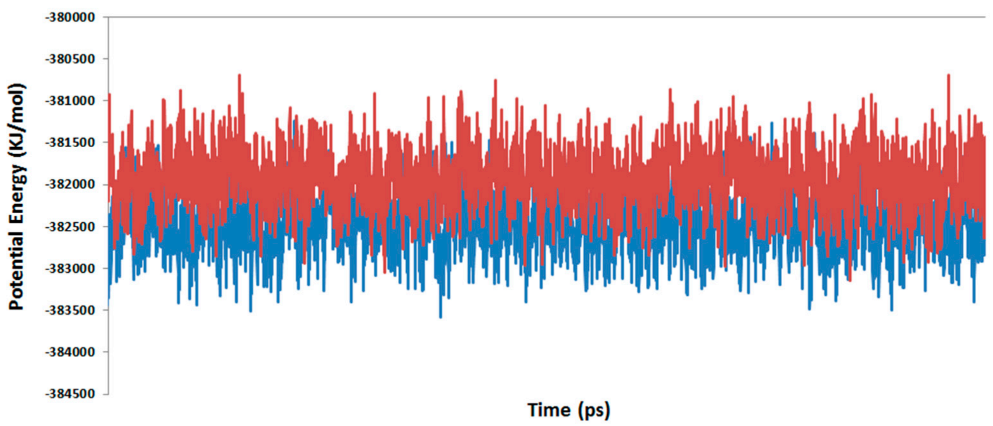

Figure S2. Time evolution of the potential energy of the full system in the wild-type (blue line) and mutated enzyme (red line).

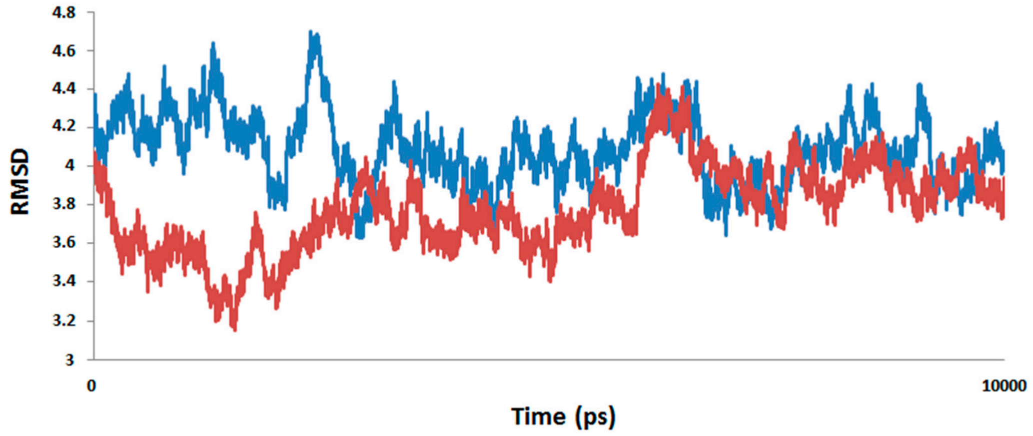

(**a**)

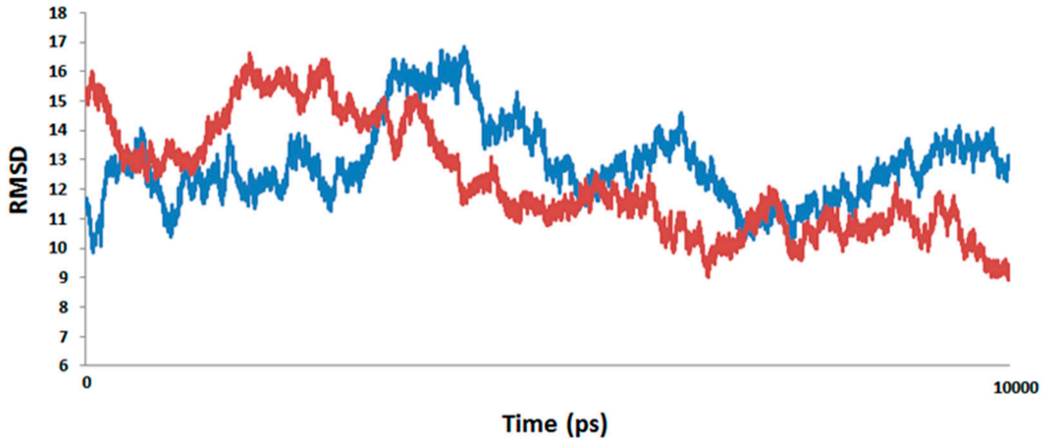

(**b**)

Figure S3. (**a**) RMSD of the carbon-alpha atoms of the protein in the wild type (blue line) and in the mutated enzyme (red line); (**b**) RMSD of the phosphorus atoms of poly-P along the last 5 ns of MD simulation in the wild type (blue line) and mutated enzyme (red line).

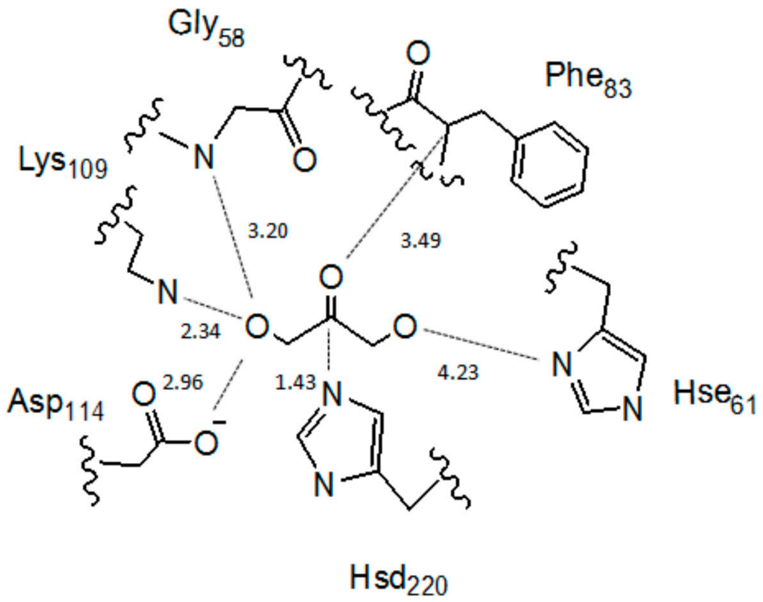

(**a**)

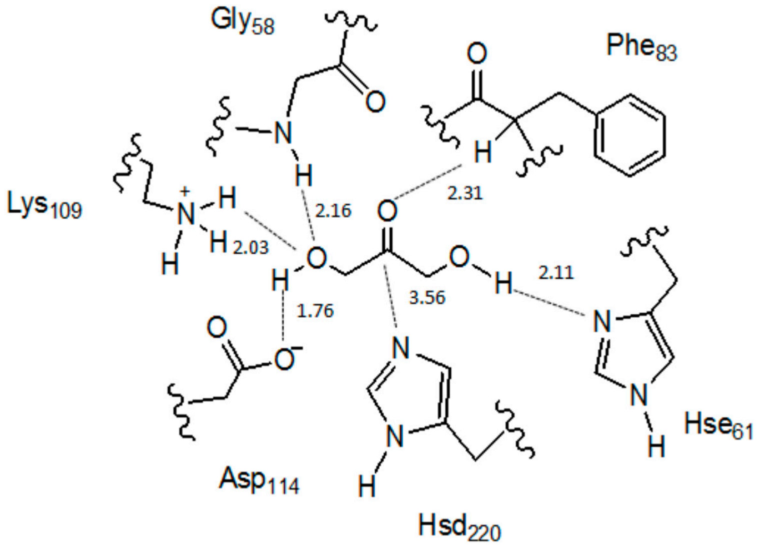

(**b**)

Figure S4. Schematic representation of the active site in the initial X-ray diffraction structure 1UN9 (**a**); and in the wild-type after 10 ns of MD simulations (**b**). Key distances between DHA and the residues of the active site are reported in Å.
